# Is remission of depressive symptoms in primary care a realistic goal? A meta-analysis

**DOI:** 10.1186/1471-2296-5-19

**Published:** 2004-09-07

**Authors:** Marliese Y Dawson, Erin E Michalak, Paul Waraich, J Ellen Anderson, Raymond W Lam

**Affiliations:** 1 Division of Clinical Neuroscience, Department of Psychiatry, University of British Columbia, 2255 Wesbrook Mall, Vancouver, BC, Canada V6T 2A1; 2 Division of Mental Health Services, Department of Psychiatry, Universityof British Columbia, 2250 Wesbrook Mall, Vancouver, BC, Canada V6T 1W6; 3 Family Physician, 6625B Sooke Road, Sooke, BC, Canada V0S 1N0

## Abstract

**Background:**

A primary goal of acute treatment for depression is clinical remission of symptoms. Most meta-analyses of remission rates involve randomized controlled trials (RCTs) using patients from psychiatric settings, but most depressed patients are treated in primary care. The goal of this study was to determine remission rates obtained in RCTs of treatment interventions for Major Depressive Disorder (MDD) conducted in primary care settings.

**Methods:**

Potentially relevant studies were identified using computerized and manual search strategies up to May 2003. Criteria for inclusion included published RCTs with a clear definition of remission using established outcome measures.

**Results:**

A total of 13 studies (N = 3202 patients) meeting inclusion criteria were identified. Overall remission rates for active interventions ranged between 50% and 67%, compared to 32% for pill placebo conditions and 35% for usual care conditions.

**Conclusions:**

Remission rates in primary care studies of depression are at least as high as for those in psychiatric settings. It is a realistic goal for family physicians to target remission of symptoms as an optimal outcome for treatment of depression.

## Background

Major depressive disorder (MDD) is one of the most common and disabling of medical conditions [1]. The Canadian Community Health Survey recently reported a one-year prevalence rate of 4.5% for MDD, indicating that over 1.1 million Canadians suffer significant distress and impairment in function due to depression [2]. The economic costs of depression are estimated at over $5 billion annually [3]. Depression is currently the fourth-ranked medical condition contributing to global burden of disease, and is estimated to rise to second overall by the year 2010 [4].

There are many effective treatments for MDD, including psychotherapy and antidepressants. Traditionally, efficacy in randomized controlled trials (RCTs) for depression has been determined on the basis of score changes in rating scales such as the Hamilton Depression Rating Scale (HDRS) [5] or the Montgomery-Asberg Depression Rating Scale (MADRS) [6]. Clinical outcome has been usually assessed by clinical response rates, typically defined as a 50% or greater reduction from baseline scores on these rating scales [7]. Although obtaining clinical response represents an important therapeutic milestone, it does not necessarily indicate a complete recovery from MDD, since many patients with clinical response will still be left with substantial residual symptoms of depression. Studies have shown that the presence of residual symptoms after an episode of MDD is associated with higher risk of relapse, recurrence, chronicity, suicide, development of cardiovascular disease, and poor quality of life [8-10].

Such findings suggest that the goals of acute treatment (approximately the first 8–12 weeks or so of treatment) for MDD should be clinical remission, a clinical state distinguished by minimal residual symptoms, rather than just response [11-13]. Clinical remission is typically defined as a score within the normal range on a given outcome measure (e.g., 17-item HDRS score of 7 or less; MADRS score of 12 or less; Clinical Global Impression [CGI] [14] severity score of "Normal, not at all ill"), although there is still some uncertainty as to the validity of these cutoff scores for symptom remission [15]. The achievement of remission is of considerable clinical importance as it predicts decreased risk of relapse and greater psychosocial functioning than typically observed in patients who have achieved clinical response alone [16-18]. Clinical remission is now identified and promoted as a clinical target for successful management of MDD in many clinical practice guidelines [13,19-21].

Increasing numbers of treatment studies are now explicitly reporting both clinical response and remission rates in assessment of outcome. A meta-analysis of 8 antidepressant studies of venlafaxine versus selective serotonin reuptake inhibitors [SSRIs] and placebo reported mean remission rates of 45%, 35%, and 25%, respectively [22]. A subsequent meta-analysis of 32 RCTs comparing venlafaxine, SSRIs and other antidepressants reported a mean overall remission rate of 42% [23]. Finally, a meta-analysis of 6 RCTs comparing antidepressants and psychotherapy in patients with MDD reported mean remission rates of 46% for each treatment [24].

All the studies in these systematic reviews involved patients in psychiatric or mixed settings. However, most people suffering from MDD will be managed in the primary care setting [25]. Approximately 5% to 10% of all patients consulting a general practitioner have MDD, with prevalence estimates being two to three times higher when other depressive disorders (i.e., minor depression or dysthymia) are included [26]. It remains unclear whether the remission rates reported in psychiatric settings can be extrapolated to primary care environments, although it is of clinical importance for primary care physicians to know whether obtaining remission is a realistic goal for their patients. There has been a recent surge in studies assessing a variety of treatment interventions for depression in primary care settings, making this an opportune time to perform a meta-analysis to address this question. Hence, the primary objective of this study was to determine remission rates obtained in RCTs of treatment interventions for MDD conducted in primary care settings.

## Methods

Potentially relevant studies were identified using computerized and manual search strategies. The computerized search conducted in June, 2003 included the databases: Medline, Psych Info, Embase, Biosis, Cochrane Database of Systematic Reviews, and Cochrane Controlled Trials Register and Current Controlled Trials (1981–May 2003). The search terms used were 'depressive disorder' or 'depression' combined with 'primary care' and 'remission' and/or variants. The bibliographies of relevant articles were also manually searched. Two reviewers (MYD and RWL) collected and independently assessed abstracts for inclusion criteria. Disagreements were resolved with consensus.

### Inclusion criteria

Studies were included if they were RCTs with original data comparing one or more interventions (e.g., antidepressant vs. cognitive behavioral therapy) and published in English. Only studies of predominantly adult populations, as opposed to exclusively child or elderly patient populations, were included. Although the focus was principally upon patients with MDD (studies primarily dealing with minor depression and dysthymia were excluded), the criteria for a diagnosis of MDD was intentionally broad in order to capture the heterogeneity of the sample and allow the results to be as generalizable as possible. Included studies also had to use a standardized outcome measure (e.g., HDRS, MADRS, Beck Depression Inventory [BDI] [27]) and provide explicit criteria for remission. While the definition of remission varied among the studies (Table 1), for the purpose of this meta-analysis we accepted each study's definition of remission, which usually was a score within the normal range on the outcome measure.

**Table 1:** Summary of included studies in meta-analysis of remission rates.

**Study**	**Diagnostic Criteria**	**Follow up Period**	**Remission Criteria**	**Total N**	**Intervention**	**Intervention Remission Rate**	**Remission %**
**Psychological Intervention Only**							
Dowrick et al., 2000 [31]	DSM-IV criteria for MDD or Adjustment Disorder	6 months	No MDD detected by SCAN interview	425	• PST • Usual Care •Prevention course	• 58/128 • 76/189 • 44/108	• 45 • 38 • 41
**Antidepressant Intervention Only**							
Benkert et al., 2000 [32]	DSM-IV criteria for MDD and HDRS ≥ 18	6 weeks	HDRS ≤ 7	275	• Mirtazapine • Paroxetine	• 52/139 • 42/136	• 37 • 31
Patris et al.,1996 [33]	DSM-IIIR criteria for MDD	8 weeks	MADRS ≤ 12	357	• Citalopram • Fluoxetine	• 114/173 • 110/184	• 66 • 60
Wade et al., 2002 [34]	DSM-IV criteria for MDD	8 weeks	MADRS ≤ 12	380	• Escitalopram • Placebo	• 92/191 • 64/189	• 48 • 34
**Psychological Intervention + Antidepressants**							
Chilvers et al., 2001 [35]	Diagnosed as MDD by GP	12 months	RDC <4, BDI <10, or clear documentation in GP notes that patient is well	103	Randomised only: • Antidepressant • Counselling	• 39/51 • 33/52	• 76 • 63
Mynors-Wallis et al., 1995 [36]	Diagnosed as MDD by GP	12 weeks	HDRS ≤ 7 or BDI ≤ 8	91	• PST • Amitriptyline • Placebo	• 18/30 • 16/31 • 8/30	• 60 • 52 • 27
Mynors-Wallis et al., 2000 [37]	RDC criteria for MDD	12 months	HDRS ≤ 8	151	• PST-group •PST-RN • Antidepressant • PST+antidepressant	• 24/39 • 23/41 • 20/36 • 23/35	• 62 • 56 • 56 • 66
Schulberg et al., 1998 [38]	DSM-IIIR criteria for MDD	8 months	HDRS ≤ 7	184	• IPT • Nortriptyline	• 49/93 • 52/91	• 57 • 53
Scott et al., 1992 [39]	DSM-IIIR criteria for MDD	4 months	HDRS ≤ 7	121	• CBT • Counselling • Amitriptyline • Usual care	• 12/30 • 22/30 • 18/31 • 14/30	• 40 • 73 • 58 • 47
**Program Interventions**							
Katon et al.,1999 [40]	Diagnosed as MDD by GP	6 months	Presence of 0 or 1 SCID-assessed symptoms	228	• Collaborative care • Usual Care	• 50/114 • 35/114	• 44 • 31
Katzelnick et al., 2000 [41]	Diagnosed as MDD by GP and HDRS ≥ 15	12 months	HDRS ≤ 7	407	• Depression management • Usual care	• 92/218 • 49/189	• 42 • 26
Kutcher et al., 2002 [42]	Diagnosed as MDD by GP	29 weeks	8 weeks or longer with HDRS ≤ 10	269	• Sertraline • Sertraline + adherence program	• 84/138 • 88/131	• 61 • 67
Rost et al., 2002 [43]	Diagnosed as MDD by GP	24 months	CES-D ≤ 16	211	• Enhanced depression care • Usual care	• 85/115 • 39/96	• 74 • 41

(Abbreviations: BDI – Beck Depression Inventory, CBT – Cognitive Behavioural Therapy, CES-D – Centre for Epidemiological Studies – Depression Scale, HDRS – Hamilton Depression Rating Scale, HSCL-D-20 – 20-item Hopkins Symptom Check List, IPT-Interpersonal Psychotherapy, MADRS – Montgomery-Asberg Depression Rating Scale, MDD – Major Depressive Disorder, PST – Problem Solving Therapy, PST-PC – Problem Solving Therapy, administered by Primary Care Physician, PST-RN – Problem Solving Therapy, administered by Registered Nurse, RDC – Research Diagnostic Criteria, SCAN – Schedules of Clinical Assessment in Neuropsychiatry, SCID – Structured Clinical Interview for DSM-III-R.)

### Data extraction

Two independent reviewers (MYD and EEM) extracted data from studies using a checklist developed for this study, with disagreements resolved by a third reviewer (RWL). A conservative measure of remission rate was calculated from each study using an intent-to-treat analysis [28], even if this method was not used in the study. For example, some studies calculated remission rates using only patients who returned for one follow-up visit post-randomization, or who had completed a course of treatment. The denominator used for remission rate was the total number of patients randomized to treatment, whether or not they were counted in the ensuing analysis. The numerator was the number of patients in remission reported in the study, regardless of the denominator used in the study analysis.

The type of intervention was classified as placebo, "usual care" by clinician (standard treatment by a patient's own physician), psychotherapy treatment only, antidepressant treatment only, psychotherapy plus antidepressant treatment, or program intervention (e.g., collaborative care using other health professionals; educational programs targeted at quality improvement for prescribing practices).

### Statistics

Each set of rates was pooled based on a Bayesian approach to meta-analysis using the Fastpro software program (version 1.7) by Eddy and Hasselblad. Readers interested in a more detailed discussion of this approach should refer to Eddy et al [29]. The pooled means and confidence intervals were calculated using Jeffrey's prior and a random effects model.

## Results

The initial electronic and bibliographic search found 63 articles of which 47 warranted more detailed review based on the published abstract. Of these, 34 articles were excluded due to methodology (not RCTs, 4 studies), lack of remission criteria (18 studies), diagnostic criteria (not MDD, 11 studies) and age criteria (geriatric, 4 studies) (some studies were excluded for multiple reasons, see Additional File 1). A final count of 13 studies met the full inclusion criteria (Table 1). In total, 3202 primary care outpatients (75% female, 25% male) were included in the analysis. The mean age of the participants was 32.1 years (range 18–73 years). The average length of follow-up was 32 weeks (range 6–104 weeks).

**Figure 1:**
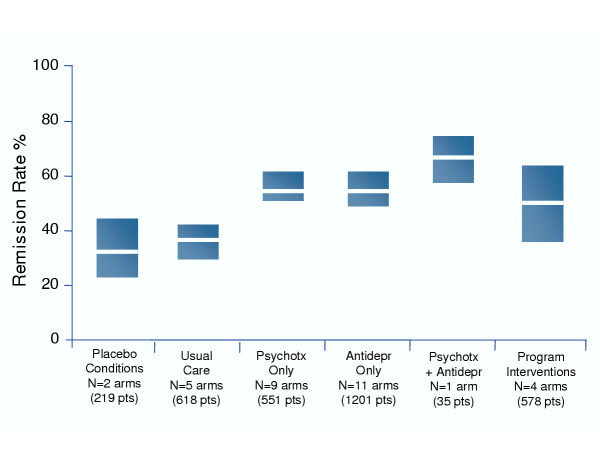
Remission rates for specific treatment conditions from randomized controlled trials (RCTs) of interventions for depression in primary care settings. The white lines represent the mean remission rates and the boxes represent the 95% confidence interval. N is the number of treatment arms in the RCTs (Note: Psychotx = Psychotherapy, Antidepr = Antidepressants, pts = patients).

The study interventions and methodologies were too heterogeneous to allow for a meaningful statistical comparison of results between treatments. Figure 1 shows mean remission rates for specific interventions. Overall remission rates for active interventions, regardless of type, ranged between 50% and 67%, compared to 32% for pill placebo conditions and 35% for usual care conditions. There were a sufficient number of antidepressant arms in the studies to permit the summary of remission rates by duration of follow-up period. For antidepressant studies with follow-up of 6 months or less, mean remission rate was 51.4% (95% C.I., 43.1%–59.6%); for antidepressant studies with greater than 6 months of follow-up, mean remission rate was 62.3% (95% C.I., 48.9%–74.8%).

## Discussion

This review of research assessing remission of depressive symptoms in primary care populations identified 13 studies meeting the inclusion criteria. Overall remission rates (regardless of type of intervention but excluding placebo or usual care arms) ranged between 50% and 67%. These rates are equivalent to, or indeed greater than, those reported in meta-analyses of studies examining pharmacological or psychological interventions for depression in psychiatric populations, in which the overall remission rates ranged between 35% and 46% [22-24]. On the one hand, we might have predicted this finding as studies conducted in primary care settings tend to include more patients with mild to moderate depression (although we excluded studies that focused exclusively upon minor depression or dysthymia), whereas patients referred to psychiatric settings are more likely to have moderate to severe depression. Primary care treatment trials also tend to be longer, favouring a higher remission rate; whereas the mean follow-up period of studies included in the current analysis was 9 months, it was only 7 weeks and 10 weeks in the two previous meta-analyses of pharmacological interventions for MDD [22,23], and 16 weeks in the meta-analysis of antidepressant versus psychotherapeutic interventions [24]. Conversely, we might have predicted that we would observe *lower *remission rates in the current meta-analysis as it included a number of studies with more lenient exclusion criteria than typically used in psychiatric clinical trials. In particular, the program intervention studies tend to include more heterogeneous patient populations as they do not routinely exclude patients with psychiatric or medical comorbidities, factors that may lessen the likelihood of obtaining remission of depressive symptoms [30].

While it was not within the scope of the current study to compare the effectiveness of different treatment interventions in improving remission rates, we can report on the trends we observed in the data. Antidepressant and psychotherapy interventions delivered in isolation showed similar remission rates (54% for both). Combination antidepressant plus psychotherapy interventions showed somewhat higher rates (67%), although this category included only 1 arm with only 35 patients. Program interventions had a mean remission rate of 50%, and all treatment interventions fared better than either placebo (32%) or usual care (35%).

The studies identified in our review were quite heterogeneous in nature, ranging from those that looked solely at the effects of a particular pharmacological agent, through to complex program initiatives that incorporated a variety of interventions at different levels of care. This heterogeneity limits our ability to make broad comments about remission rates in primary care, but was not unexpected, as we wanted to capture the diversity of treatment interventions for depression currently being tested in this setting. Other potential limitations of the study include that fact that we only assessed published studies written in English and that we used a conservative measure of remission rate. Finally, we also used the definition of remission as specified by each individual study. While these definitions were similar to those widely used in RCTs conducted in psychiatric settings, and thus are useful for comparison, there is current controversy about depression scales and which cutoff scores indicate true remission of symptoms [15].

## Conclusions

This meta-analysis serves to answer an important clinical question about the feasibility of obtaining remission of symptoms of MDD in primary care patients. Our results indicate that this is a realistic goal in this population, although further research is still required to determine whether certain treatment modalities (or combinations of treatment interventions) are superior to others in achieving higher remission rates. Future research should also focus upon developing pragmatic strategies for general practitioners to implement evidence-based guidelines concerning the treatment of depression to clinical remission.

## Authors' contributions

MYD and EEM conducted the data extraction, wrote the initial draft of the manuscript, interpreted results, and revised the manuscript. PW provided statistical consultation and analysis, and revised the manuscript. JEA interpreted the results and revised the manuscript. RWL conceived the initial idea, developed the method, interpreted results, revised the manuscript, and provided financial resources for the study. All authors read and approved the final manuscript.

## Competing interests

RWL is on advisory/speaker boards or has received research funds from: AstraZeneca, Biovail, Canadian Network for Mood and Anxiety Treatments, Eli Lilly, GlaxoSmithKline, Janssen-Ortho, Litebook, Inc., Lundbeck, Merck, Organon, Roche, Shire, Servier, and Wyeth.

## Pre-publication history

The pre-publication history for this paper can be accessed here:



## Supplementary Material

Additional File 1 Studies excluded from the review Click here for file
