# Supplementary material for: Is remission of depressive symptoms in primary care a realistic goal? A meta-analysis
Source: BMC Fam Pract. 2004 Sep 7;5:19. doi: 10.1186/1471-2296-5-19 (PMC518964; doi:10.1186/1471-2296-5-19)
Supplement: Additional File 1 — Studies excluded from the review [file 1471-2296-5-19-S1.pdf]

Studies excluded from the review

Papers excluded from analysis	Reason for exclusion
Barrett JE, Williams JW, Jr., Oxman TE, Frank E, Katon W, Sullivan M, Hegel MT, Cornell JE, Sengupta AS: Treatment of dysthymia and minor depression in primary care: a randomized trial in patients aged 18 to 59 years. J Fam Pract 2001, 50: 405-412.	Not MDD
Bedi N, Chilvers C, Churchill R, Dewey M, Duggan C, Fielding K, Gretton V, Miller P, Harrison G, Lee A, Williams I: Assessing effectiveness of treatment of depression in primary care. Partially randomised preference trial. Br J Psychiatry 2000, 177: 312-318.	Not RCT
Bouchard JM, Delaunay J, Delisle JP, Grasset N, Mermberg PF, Molczadzki M, Pagot R, Richou H, Robert G, Ropert R, .: Citalopram versus maprotiline: a controlled, clinical multicentre trial in depressed patients. Acta Psychiatr Scand 1987, 76: 583-592.	Unable to determine if patients came from primary care population
Boudreau DM, Capoccia KL, Sullivan SD, Blough DK, Ellsworth AJ, Clark DL, Katon WJ, Walker EA, Stevens NG: Collaborative care model to improve outcomes in major depression. Ann Pharmacother 2002, 36: 585-591.	Remission rates not included
Bougerol T, Scotto JC, Patris M, Strub N, Lemming S, Hopfner Peterson HE. Citalopram and fluoxetine in major depression. Comparison of two clinical trials in a psychiatrist setting and in general practice. Clinical Drug Investigation 14[2], 77-89. 1997.	2 studies, one of which is a repeat of the Patris article which is included in analysis
Browne G, Steiner M, Roberts J, Gafni A, Byrne C, Dunn E, Bell B, Mills M, Chalklin L, Wallik D, Kraemer J: Sertraline and/or interpersonal psychotherapy for patients with dysthymic disorder in primary care: 6-month comparison with longitudinal 2-year follow-up of effectiveness and costs. J Affect Disord 2002, 68: 317-330.	Not MDD; remission rates not included
Coyne JC, Klinkman MS, Gallo SM, Schwenk TL: Short-term outcomes of detected and undetected depressed primary care patients and depressed psychiatric patients. Gen Hosp Psychiatry 1997, 19: 333-343.	Not RCT
Finley PR, Rens HR, Pont JT, Gess SL, Louie C, Bull SA, Bero LA: Impact of a collaborative pharmacy practice model on the treatment of depression in primary care. Am J Health Syst Pharm 2002, 59: 1518-1526.	Remission rates not included
Frank E, Rucci P, Katon W, Barrett J, Williams JW, Jr., Oxman T, Sullivan M, Cornell J: Correlates of remission in primary care patients treated for minor depression. Gen Hosp Psychiatry 2002, 24: 12-19.	Not MDD

Hedrick SC, Chaney EF, Felker B, Liu CF, Hasenberg N, Heagerty P, Buchanan J, Bagala R, Greenberg D, Paden G, Fihn SD, Katon W: Effectiveness of collaborative care depression treatment in Veterans' Affairs primary care. J Gen Intern Med 2003, 18: 9-16.	Not MDD; remission rates not included
Hunkeler EM, Meresman JF, Hargreaves WA, Fireman B, Berman WH, Kirsch AJ, Groebe J, Hurt SW, Braden P, Getzell M, Feigenbaum PA, Peng T, Salzer M: Efficacy of nurse telehealth care and peer support in augmenting treatment of depression in primary care. Arch Fam Med 2000, 9: 700-708.	Remission rates not included
Jacob KS, Bhugra D, Mann AH: A randomised controlled trial of an educational intervention for depression among Asian women in primary care in the United Kingdom. Int J Soc Psychiatry 2002, 48: 139-148.	Not MDD
Katon W, Von KM, Lin E, Walker E, Simon GE, Bush T, Robinson P, Russo J: Collaborative management to achieve treatment guidelines. Impact on depression in primary care. JAMA 1995, 273: 1026-1031.	Remission rates not included
Katon W, Robinson P, Von KM, Lin E, Bush T, Ludman E, Simon G, Walker E: A multifaceted intervention to improve treatment of depression in primary care. Arch Gen Psychiatry 1996, 53: 924-932.	Remission rates not included
Kroenke K, West SL, Swindle R, Gilsenan A, Eckert GJ, Dolor R, Stang P, Zhou XH, Hays R, Weinberger M: Similar effectiveness of paroxetine, fluoxetine, and sertraline in primary care: a randomized trial. JAMA 2001, 286: 2947-2955.	Remission rates not included
Lin EH, Simon GE, Katon WJ, Russo JE, Von KM, Bush TM, Ludman EJ, Walker EA: Can enhanced acute-phase treatment of depression improve long-term outcomes? A report of randomized trials in primary care. Am J Psychiatry 1999, 156: 643-645.	Follow-up assessment to the two Katon studies; remission rates not included
Lin EH, VonKorff M, Russo J, Katon W, Simon GE, Unutzer J, Bush T, Walker E, Ludman E: Can depression treatment in primary care reduce disability? A stepped care approach. Arch Fam Med 2000, 9: 1052-1058.	Remission rates not included
Malt UF, Robak OH, Madsbu HP, Bakke O, Loeb M: The Norwegian naturalistic treatment study of depression in general practice (NORDEP)-I: randomised double blind study. BMJ 1999, 318: 1180-1184.	Remission rates not included
Mann AH, Blizard R, Murray J, Smith JA, Botega N, MacDonald E, Wilkinson G: An evaluation of practice nurses working with general practitioners to treat people with depression. Br J Gen Pract 1998, 48: 875-879.	Not MDD

Peveler R, George C, Kinmonth AL, Campbell M, Thompson C: Effect of antidepressant drug counselling and information leaflets on adherence to drug treatment in primary care: randomised controlled trial. BMJ 1999, 319: 612-615.	Remission rates not included
Ravindran AV, Anisman H, Merali Z, Charbonneau Y, Telner J, Bialik RJ, Wiens A, Ellis J, Griffiths J: Treatment of primary dysthymia with group cognitive therapy and pharmacotherapy: clinical symptoms and functional impairments. Am J Psychiatry 1999, 156: 1608-1617.	Not MDD; remission rates not included
Schmaling KB, Dimidjian S, Katon W, Sullivan M: Response styles among patients with minor depression and dysthymia in primary care. J Abnorm Psychol 2002, 111: 350-356.	Not MDD
Schweizer E, Rickels K, Hassman H, Garcia-Espana F: Buspirone and imipramine for the treatment of major depression in the elderly. J Clin Psychiatry 1998, 59: 175-183.	Geriatric population
Simon GE, VonKorff M, Rutter C, Wagner E: Randomised trial of monitoring, feedback, and management of care by telephone to improve treatment of depression in primary care. BMJ 2000, 320: 550-554.	Remission rates not clearly reported
Simpson S, Corney R, Fitzgerald P, Beecham J: A randomized controlled trial to evaluate the effectiveness and cost-effectiveness of psychodynamic counselling for general practice patients with chronic depression. Psychol Med 2003, 33: 229-239.	Not MDD; remission rates not included
Sullivan MD, Katon WJ, Russo JE, Frank E, Barrett JE, Oxman TE, Williams JW, Jr.: Patient beliefs predict response to paroxetine among primary care patients with dysthymia and minor depression. J Am Board Fam Pract 2003, 16: 22-31.	Not MDD
Taragano F, Lyketsos CG, Paz J, Schapira M, Comesana DE, Klimovsky S: An open-label trial of sertraline for the treatment of major depression in primary care. Ann Clin Psychiatry 1999, 11: 67-71.	Not RCT
Thomas L, Mulsant BH, Solano FX, Black AM, Bensasi S, Flynn T, Harman JS, Rollman BL, Post EP, Pollock BG, Reynolds CF, III: Response speed and rate of remission in primary and specialty care of elderly patients with depression. Am J Geriatr Psychiatry 2002, 10: 583-591.	Geriatric population
Thompson C, Peveler RC, Stephenson D, McKendrick J: Compliance with antidepressant medication in the treatment of major depressive disorder in primary care: a randomized comparison of fluoxetine and a tricyclic antidepressant. Am J Psychiatry 2000, 157: 338-343.	Remission rates not included

Unutzer J, Katon W, Williams Jr JW, Callahan CM, Harpole L, Hunkeler EM, Hoffing M, Arean P, Hegel MT, Schoenbaum M, Oishi SM, Langston CA: Improving primary care for depression in late life the design of a multicenter randomized trial. Med Care 2001, 39: 785-799.	Geriatric population
Unutzer J, Katon W, Callahan CM, Williams JW, Jr., Hunkeler E, Harpole L, Hoffing M, Della Penna RD, Noel PH, Lin EH, Arean PA, Hegel MT, Tang L, Belin TR, Oishi S, Langston C: Collaborative care management of late-life depression in the primary care setting: a randomized controlled trial. JAMA 2002, 288: 2836-2845.	Geriatric population
Wells KB, Sherbourne C, Schoenbaum M, Duan N, Meredith L, Unutzer J, Miranda J, Carney MF, Rubenstein LV: Impact of disseminating quality improvement programs for depression in managed primary care: a randomized controlled trial. JAMA 2000, 283: 212-220.	Remission rates not included; quasi RCT
Williams JW, Jr., Barrett J, Oxman T, Frank E, Katon W, Sullivan M, Cornell J, Sengupta A: Treatment of Dysthymia and Minor Depression in Primary Care: A Randomized Controlled Trial in Older Adults. JAMA 2000, 284: 1519-1526.	Not MDD
Worrall G, Angel J, Chaulk P, Clarke C, Robbins M: Effectiveness of an educational strategy to improve family physicians' detection and management of depression: a randomized controlled trial. CMAJ 1999, 161: 37-40.	Remission rates not included
